# Evaluation of the contextualized sexual and reproductive health educational strategy “Rurankapak”: a mixed-methods quasi-experimental study among adolescents and young people in Ecuador

**DOI:** 10.3389/frph.2026.1783094

**Published:** 2026-02-19

**Authors:** Jeny Valencia, Jesús Endara-Mina, Andrea Morales, Andrea Huertas, Domenica Espinosa, Johao Sinchiguano, María-José Martínez, Santiago Negrete, Yesenia Cacuango, Jefferson Ortega, Paulina Rios-Quituizaca

**Affiliations:** 1 Facultad de Ciencias Médicas, Carrera de Obstetricia, Universidad Central del Ecuador (UCE), Quito, Ecuador; 2 Facultad de Ciencias Jurídicas y Políticas, Universidad Técnica Particular de Loja (UTPL), Loja, Ecuador; 3 Coordinación de Docencia e Investigación, Hospital Provincial General Pablo Arturo Suárez, Quito, Ecuador

**Keywords:** indigenous peoples, reproductive health, Rurankapak, sexual education, sexual health

## Abstract

**Background:**

Sexual and reproductive health (SRH) educational interventions in Ecuador show uneven effectiveness, particularly among adolescents and young people in socioculturally diverse settings. Although the “Rurankapak” methodology has been implemented as a participatory SRH education strategy, its standardized application has limited cultural relevance, acceptability, and sustainability.

**Objectives:**

To collaboratively adapt the Rurankapak methodology for SRH education to diverse sociocultural contexts and to preliminarily assess its applicability and acceptability among adolescents and young people in Ecuador.

**Methods:**

A mixed-methods quasi-experimental study was conducted in three phases. The qualitative phase included semi-structured interviews with key informants experienced in Rurankapak implementation, analyzed using thematic coding. Findings informed a participatory redesign process involving Obstetrics students trained as peer facilitators. The quantitative phase followed a before–after design, applying the adapted intervention to four population groups and administering pre–post knowledge assessments (Cronbach's *α* = 0.81) and satisfaction surveys.

**Results:**

Qualitative findings identified the need to strengthen pre-workshop planning, referral pathways, and the management of complex topics. An adapted version of Rurankapak was developed, structured into seven educational stations with culturally tailored materials. Quantitative results showed high acceptability among adolescents and young people, with lower acceptance among parents. Peer facilitators demonstrated strong pedagogical performance and reported improvements in communication skills, empathy, and intercultural competence.

**Conclusions:**

Contextual adaptation and participatory redesign enhance the cultural relevance and feasibility of SRH educational strategies. The use of peer facilitators supports adolescent engagement and contributes to the comprehensive training of future SRH professionals.

## 1 Introduction

Sustainable Development Goal (SDG) 3.7 establishes that by 2030, States must ensure universal access to sexual and reproductive health (SRH) services, including family planning, comprehensive education, and the integration of SRH into national programs (1). International organizations such as the World Health Organization (WHO), the United Nations Children's Fund (UNICEF), and the Lancet Commission emphasize the urgent need to prioritize adolescents in achieving the SDGs (2).

Despite global commitments, adolescent SRH remains a critical challenge, particularly in rural areas and among vulnerable populations. In Ecuador, according to the National Health and Nutrition Survey (ENSANUT) 2018, 98.3% of women aged 15–24 reported having received information on SRH; however, the persistently high rate of adolescent pregnancy—2 live births per 1,000 girls aged 10–14 in 2022—reveals a gap between knowledge and practice (3). Moreover, 50.9% of adolescents hold negative attitudes toward sexually transmitted infections (STIs), 40.6% report engaging in risk behaviors, and 8% have experienced an STI, highlighting deficiencies in prevention and access to services (4–6). Across Latin America, social, cultural, and religious barriers, as well as gender-based violence, constrain the free exercise of sexuality, particularly among Indigenous peoples and rural populations (7). These groups face structural inequities in education and health, compounded by discrimination and machismo, which hinder the development of life projects. Consequently, culturally appropriate educational strategies are required to address these challenges effectively.

The implementation of Comprehensive Sexuality Education (CSE) has followed diverse trajectories, with heterogeneous regulatory frameworks and varying degrees of institutionalization. Countries such as Argentina, Uruguay, and Cuba have made substantial progress through specific programs and legislation recognizing CSE as a cross-cutting right, integrating it into school curricula and teacher training. In contrast, countries such as Peru, the Dominican Republic, and Honduras lack robust regulatory frameworks, limiting reach and allowing religious and familial beliefs to exert substantial influence over educational content (8). In Ecuador, qualitative research has shown that sexuality education in schools tends to be unsystematic, fragmented, and frequently silenced. Adolescent parents report that teachers often lack adequate preparation and address topics through a biologicist and moralizing lens, restricting open dialogue and the inclusion of emotional, relational, and gender dimensions (7).

Although information on SRH appears to be widely available, there is a pressing need to strengthen contextualized educational methodologies that promote informed decision-making regarding sexuality, STI prevention, and unplanned pregnancies (9). The CSE framework promoted by the United Nations Educational, Scientific and Cultural Organization (UNESCO) emphasizes teaching and learning across the cognitive, emotional, physical, and social dimensions of sexuality, adapted to age and developmental stage, and underscores the importance of linking knowledge with access to essential SRH services (1). Evaluating the effectiveness of such interventions is crucial to informing evidence-based public policies.

Experiences in Latin America have demonstrated that educational programs led by midwives can significantly reduce adverse SRH indicators (10). In Ecuador, these professionals receive specialized training in prevention and community health and are more prevalent in rural settings; however, their role remains insufficiently recognized within national programs. Within this context, the educational strategy “Rurankapak” (11), promoted by the United Nations Population Fund (UNFPA) since 2012 in several Amazonian provinces (Sucumbíos and Orellana) and subsequently expanded nationwide, aims to promote sexual and reproductive rights, prevent adolescent pregnancy and gender-based violence, and integrate nutrition. Despite its national implementation and inclusion in facilitator training across disciplines, further studies are needed to assess the effectiveness of brief interventions across diverse cultural contexts, particularly considering the potential of trained university students as peer facilitators.

From a conceptual standpoint, this study is grounded in a rights-based approach to sexual and reproductive health education, which frames access to comprehensive sexuality education as an integral component of the right to health, education, and information. International frameworks emphasize that effective SRH education must be accessible, acceptable, and culturally appropriate, particularly for populations experiencing structural inequities, such as Indigenous and rural communities (12, 13). In intercultural contexts, this approach requires the recognition of adolescents and young people as active subjects of rights, as well as the adaptation of educational content to local sociocultural realities, languages, and worldviews. Such perspectives are consistent with international recommendations highlighting the importance of culturally responsive and participatory educational strategies to reduce health inequities and promote adolescent agency in sexual and reproductive decision-making (14, 15).

In addition, the study is informed by principles of critical pedagogy and participatory education, which emphasize dialogue, reflection, and knowledge co-construction as central mechanisms for meaningful learning (16). These principles are closely aligned with experiential learning theory, which posits that knowledge acquisition is enhanced when learners actively engage with content through experience, reflection, and application (17). Within sexual and reproductive health education, such approaches have been operationalized through peer education models, where trained peers facilitate learning processes, reduce generational barriers, and foster environments of trust and openness. Evidence suggests that peer-led and experiential educational interventions can improve knowledge, communication skills, and engagement among adolescents and young people, particularly in culturally diverse settings (18, 19).

The present study sought to collaboratively redesign the “Rurankapak” methodology for SRH education, adapting it to the sociocultural characteristics of adolescents and young people, and to preliminarily evaluate its applicability and acceptability.

## 2 Methods

### 2.1 Study design

The study adopted a mixed-methods, sequential, exploratory, quasi-experimental design. This approach enabled an initial qualitative phase to explore in depth the accumulated experience in implementing the “Rurankapak” methodology and, based on this evidence, to adapt the intervention and subsequently assess its applicability and acceptability using a before–after quantitative design across diverse target populations.

The mixed-methods and participatory design of the study is consistent with rights-based and critical pedagical approaches, which emphasize dialogue, contextual understanding, and the active involvement of participants in the construction of knowledge (12, 16).

### 2.2 Description of the Rurankapak educational strategy

**“**Rurankapak” (Para Hacerlo – RuranKapak) (20), is a participatory, station-based SRH educational methodology developed in Ecuador in 2012 through collaboration between civil society organizations, Indigenous community groups, and the health and education sectors. The original methodology consists of a structured participatory circuit with eight thematic stations addressing false beliefs and taboos, sexuality, menstrual health, contraceptive methods, sexual and reproductive rights, rights violations, gender-based violence, and nutrition and adolescent care.

The pedagogical approach is grounded in experiential and playful learning, using peer-based activities that promote dialogue, horizontal knowledge exchange, and respect for diversity. The methodology integrates cross-cutting perspectives of human rights, gender, interculturality, and intergenerational dialogue and has been adapted to diverse sociocultural contexts and emergency scenarios.

In this study, a systematically adapted version of Rurankapak was implemented based on qualitative findings. The adapted intervention comprised seven thematic stations with predefined learning objectives and standardized facilitation guidelines. Stations were delivered to groups of 5–7 participants, with an average duration of 12–15 minutes per station, following a standardized participatory sequence.

### 2.3 Study setting

The research was conducted within the framework of a collaboration between the Obstetrics Program of the Faculty of Medical Sciences at the Universidad Central del Ecuador (UCE), the Law Program of the Universidad Técnica Particular de Loja (UTPL), and UNFPA, the organization that developed and promotes the “Rurankapak” strategy. This interdisciplinary collaboration ensured the integration of public health, educational, and legal perspectives, particularly with regard to the protection and promotion of the rights of Indigenous peoples and nationalities. The “Rurankapak” methodology, created in 2012 through participatory processes with adolescents, has since been implemented in several provinces nationwide. The academic staff of the Obstetrics Program has incorporated “Rurankapak” into its curriculum, facilitating critical analysis, learning, and implementation across diverse settings. This experience underscored the need to evaluate the methodology in culturally differentiated contexts, including rural, urban-marginal, and Indigenous settings.

### 2.4 Participants

Three categories of participants were included: (a) key informants with expertise in implementing “Rurankapak” (professionals from health, education, and psychology), (b) Obstetrics undergraduate students selected as facilitators, and (c) workshop participants, including rural-Indigenous adolescents, adolescents from urban-marginal schools, early-year university students enrolled in health-related programs, and parents from rural communities. Experts were recruited using snowball sampling based on referrals provided by UNFPA. Facilitators were selected by convenience from final-year students with demonstrated academic leadership and availability for training. Workshop participants were recruited through local institutions supporting the study.

The use of trained university students as peer facilitators aligns with peer education and experiential learning frameworks, which highlight the value of reduced generational distance and learning through interaction and reflection (18).

### 2.5 Inclusion and exclusion criteria

Criteria were defined for each participant group to ensure relevance and homogeneity:

- Experts (key informants): inclusion criteria comprised documented experience in applying the “Rurankapak” methodology in at least one intervention; exclusion criteria included lack of direct experience with Rurankapak or experience limited to theoretical activities without field implementation.
- Facilitators (students): inclusion criteria included being enrolled in the final semester of the Obstetrics Program and availability to participate in training sessions and workshop implementation; exclusion criteria included not being in the final semester or inability to complete the training process.
- Workshop participants (recipients): inclusion criteria comprised belonging to one of the four predefined population groups, being of legal age or having assent and authorization from legal guardians in the case of minors, and voluntary participation through informed consent; exclusion criteria included prior participation in similar Rurankapak interventions within the previous 12 months (to avoid learning effects) or refusal to provide consent/assent. These criteria were applied during pre-implementation procedures and documented in recruitment records.

### 2.6 Sampling and pilot validation

Snowball sampling was used for experts, purposive sampling based on academic profile for facilitators, and convenience sampling coordinated with local institutions for workshop participants. Prior to full implementation, a pilot test of the knowledge instrument and workshop logistics was conducted with a volunteer group of university students. The pilot allowed refinement of item wording and workshop dynamics and demonstrated adequate internal consistency of the main questionnaire (Cronbach's *α* = 0.81), supporting its reliability for pre–post measurement. The validated knowledge questionnaire is provided as Supplementary Material.

As this was an exploratory, feasibility-oriented study, no *a priori* sample size calculation was performed. Sample size was determined by institutional feasibility and participant availability at each implementation site.

### 2.7 Procedure (study phases)

The study was conducted in three sequential and interrelated phases. Phase I (qualitative) involved virtual semi-structured interviews with key informants, conducted in two sessions per participant to explore cultural relevance, barriers, and recommendations; findings informed the methodological redesign. Phase II (facilitator training) included 3–4 collaborative workshops and at least two practice-simulation sessions for selected Obstetrics students, during which stations were rehearsed and structured feedback provided; learning perception and satisfaction surveys were administered. Phase III (quantitative intervention) implemented the adapted “Rurankapak” version across the four target groups; participants completed a pretest immediately before the workshop and a posttest upon completion, along with a post-intervention satisfaction survey.

Phase I (qualitative component) included six key informants interviewed through semi-structured key informant interviews. Phase II involved 16 Obstetrics undergraduate students trained as peer facilitators. Phase III included a total of 167 participants distributed across four groups: urban adolescents (*n* = 49), rural/Indigenous adolescents (*n* = 40), parents from rural communities (*n* = 27), and university students in early semesters (*n* = 51).

### 2.8 Data collection instruments and validation

Primary instruments included: (a) a semi-structured interview guide for key informants; (b) a 14-item knowledge questionnaire developed *ad hoc* for the thematic stations of Rurankapak—content-validated by expert reviewers and refined after pilot testing; (c) a satisfaction survey with Likert-scale items and open-ended questions assessing clarity, cultural relevance, and practical utility; and (d) a facilitator self-assessment survey exploring competencies, challenges, and willingness to replicate the methodology. Pilot testing allowed correction of ambiguities and confirmed internal consistency (*α* = 0.81). Paper-based instruments were administered by trained surveyors.

### 2.9 Qualitative analysis

The qualitative component consisted of six key informant interviews conducted with professionals from the health, education, and psychology sectors who had direct experience implementing the Rurankapak methodology. Interviews were semi-structured, guided by a flexible interview protocol exploring implementation experience, cultural relevance, perceived barriers, and recommendations for adaptation. All interviews were conducted virtually, lasted approximately 45–60 min, and were audio-recorded with participant consent.

Interviews were transcribed verbatim and analyzed using ATLAS.ti v22 to support coding and category organization. A thematic analysis approach was applied, including open coding, category development, and construction of semantic networks to identify patterns and adaptation recommendations. To strengthen analytical validity, independent double coding was conducted by two researchers, discrepancies were resolved through discussion and consensus, inter-coder agreement was documented, and member checking was performed with selected key participants. Triangulation across interviews, methodological documents, and observations from rehearsal sessions enhanced the credibility of qualitative findings.

### 2.10 Quantitative analysis

Quantitative data were processed using IBM SPSS Statistics version 25.0. As pre–post score distributions did not meet normality assumptions across multiple groups, the non-parametric Wilcoxon signed-rank test was applied to compare scores before and after the intervention. Statistical significance was set at *p* < 0.05, and exact p-values were reported when appropriate (e.g., preliminary analyses showed *p* ≤ 0.001 in certain subgroups; final values are presented in the Results section). In addition to *p*-values, effect size measures suitable for non-parametric tests (e.g., r derived from Z statistics) were calculated to estimate the magnitude of observed changes. Quantitative findings were integrated with qualitative evidence to assess the applicability, acceptability, and operational feasibility of the adapted “Rurankapak” version.

### 2.11 Methods integration (triangulation)

To address the study objectives, a convergent triangulation strategy was employed: qualitative findings informed methodological modifications that were operationalized and evaluated quantitatively; discrepancies between qualitative and quantitative results were explored through comparison matrices and consultations with facilitators and experts to interpret findings and refine practical recommendations.

### 2.12 Ethical considerations

This study was approved by the Human Research Ethics Committee of the Central University of Ecuador (code 004-DOC-FCM-2025). The research was conducted in accordance with the ethical principles of the World Medical Association's Declaration of Helsinki. All participants received written information regarding the study's purpose, procedures, and duration and were informed of their right to withdraw at any time without consequences. Data were used exclusively for research purposes and handled under strict confidentiality to protect participant privacy. Participants who understood and agreed to the guidelines were enrolled after providing informed consent; for minors, assent and authorization from legal guardians were obtained.

## 3 Results

### 3.1 Qualitative component

Six key informant interviews were conducted with professionals from the health, education, and psychology sectors who had direct experience implementing the Rurankapak methodology. The analysis identified recurrent themes related to methodological structure, content coverage, and contextual implementation challenges.

Key informants reported difficulties in addressing sensitive topics, particularly abortion, HIV, and sexual violence, due to sociocultural resistance and limited institutional preparedness. Participants also highlighted gaps in referral pathways and the absence of clearly defined support networks for cases involving rights violations. Additional concerns included the clarity and cultural appropriateness of certain educational materials, as well as the need for greater flexibility in adapting content to participant age and context. Based on these findings, several methodological adjustments were proposed, including strengthening pre-workshop planning, clarifying learning objectives, improving visual materials, and reinforcing facilitator training processes. The analysis generated key insights that guided the adaptation of the intervention, as summarized in Table 1.

**Table 1:** Main findings from key informant interviews.

Category	Identified subcategories and Verbatim quotations
Most sensitive or challenging topics	*Taboo topics and sociocultural resistance: “…the cultural context here, at least in the Amazon region, is terrible…” / “…the DECE staff… flatly refused to talk about the right to abortion…” / “…the abortion issue… they categorically refused to discuss the right…”*
	*Insufficient coverage of critical content: “…there is no specific explanation in the guide on this topic…” (referring to HIV and abortion)*
	*Sexual violence and lack of institutional preparedness: “…an alert was triggered due to sexual violence… the girl… was unclear about what to do…” / “…what to do if someone discloses abuse… how to support the case or know where to refer it…” / “…there is a lack of tools for facilitation… in cases of sexual violence…”*
	*Need for institutional coordination and response: “…to create a support network… to communicate it… which processes must be followed…” / “…to review and reinforce whether the health center has it and how access is guaranteed…”*
Proposed improvements or corrections	*Diagnostic tool: “…it is a gauge that later allows us to see which topics we need to explore more deeply…”*
	*Pedagogical and methodological adjustments: “…to select the cards also depending on the group or the adolescents’ age…” / “…nothing is a rigid framework. Every methodological guide provides general guidelines…”*
	*Unclear or insufficient materials: “…some images may seem a bit confusing for the adolescents…” / “…materials on sexual diversity… were not very visible…” / “…the menstrual cycle… like advanced Chinese, it's very abstract…” / “…we did not fully understand [Station 8]… we went over it several times…”*
	*Capacity strengthening: “…they should be trained… over a longer period of one or two months…”*
	*Expansion and updating of resources: “…there are myths, games, access to other links… with official information…” / “…to develop TikTok videos with accurate information…”*

DECE, *Departamento de Consejería Estudiantil* (Student Counseling Department); HIV, Human Immunodeficiency Virus.

#### 3.1.1 Station-specific improvements and facilitator training

Each station was reviewed and adapted through the development of updated instructional guidelines, including modifications to learning objectives, participatory dynamics, and the selection of materials. Audiovisual resources were incorporated, and a digital blog with key messages and embedded links was introduced as part of the adapted strategy. Content and activities were adjusted to ensure cultural and age appropriateness across target groups. As a result of this process, the Rurankapak Guide: Methodological Instruction Manual – Adapted Version was produced and internally reviewed by the academic teams of the Obstetrics Program at the Universidad Central del Ecuador (UCE) and the Law Program at the Universidad Técnica Particular de Loja (UTPL).

Facilitator training was conducted in three phases: (1) individual familiarization with the methodological guide; (2) peer-to-peer exchange sessions; and (3) supervised practice of the stations in simulated settings. Sixteen Obstetrics undergraduate students completed the full training process and subsequently participated as facilitators in the intervention.

### 3.2 Quantitative component

A total of 167 participants completed pre- and post-intervention assessments across four population groups: urban adolescents (*n* = 49), rural/Indigenous adolescents (*n* = 40), parents from rural communities (*n* = 27), and university students (*n* = 41) in early semesters of health-related programs. The sociodemographic characteristics of participants by group are presented in Table 2**.**

**Table 2:** Sociodemographic characteristics of study participants by group.

Variable	Category	Young adult group (university students) *n* = 41	Urban adolescent group (urban school) *n* = 49	Rural adolescent group (rural school) *n* = 40	Parents (rural school) *n* = 27
Age (years)	–	20.6 ± 1.4; 21.0 (18–23)	16.0 ± 0.8; 16.0 (15–18)	17.3 ± 1.3; 17.5 (15–19)	34.7 ± 2.0; 34.0 (32-41)
Sex	Female	31 (75.6%)	24 (49.0%)	22 (55.0%)	24 (88.9%)
	Male	10 (24.4%)	25 (51.0%)	18 (45.0%)	3 (11.1%)
Ethnicity	Mestizo	35 (85.4%)	39 (79.6%)	5 (12.5%)	4 (14.8%)
	White	5 (12.2%)	5 (10.2%)	–	–
	Indigenous	1 (2.4%)	2 (4.1%)	35 (87.5%)	23 (85.2%)
	Montubio	–	3 (6.1%)	–	–

Data are presented as mean ± standard deviation; median (range), or *n* (%).

The internal consistency of the knowledge questionnaire was acceptable (Cronbach's *α* = 0.81). Pre–post comparisons indicated statistically significant improvements in overall knowledge scores among university students and rural/Indigenous adolescents (Wilcoxon signed-rank test, *p* < 0.05). No statistically significant changes were observed among urban adolescents or parents. The mean age of school students was 17.3 years (SD: 1.3), while the mean age of young adults was 20.7 years (SD: 1.4). The young adult (university) group showed statistically significant post-intervention improvements across all stations (*p* = 0.001). Students from rural/Indigenous schools demonstrated significant improvements in Stations 2 and 4, related to sexuality and contraception (*p* = 0.001), as well as in total scores. No statistically significant pre–post differences were observed in the remaining groups (see Table 3).

**Table 3:** Analysis of the results of the modified Rurankapak intervention among urban and rural secondary school students, university students, and parents.

Rurankapak stations	Adolescents from an urban-marginal school			Indigenous adolescents from a rural school			Parents from a rural school			Young adult students in early semesters of health programs		
	Pretest	Posttest	*p* *	Pretest	Posttest	*p* *	Pretest	Posttest	*p* *	Pretest	Posttest	*p* *
	Mean (SD)	Mean (SD)		Mean (SD)	Mean (SD)		Mean (SD)	Mean (SD)		Mean (SD)	Mean (SD)	
False beliefs and taboos	3.73 (0.64)	3.78 (0.66)	0.82	3.97 (0.66)	4.14 (0.79)	0.21	4.06 (0.85)	4.03 (0.8)	0.87	4.01 (0.48)	4.67 (0.53)	0.000
Sexuality	2.89 (0.81)	3.1 (0.88)	0.23	2.56 (0.91)	3.45 (1.14)	0.000	2.68 (1.02)	3.15 (1.22)	0.04	3.65 (0.92)	4.15 (1.11)	0.000
Menstrual health	3.65 (0.72)	3.72 (0.64)	0.7	3.48 (0.86)	3.76 (0.92)	0.1	3.81 (0.91)	3.98 (0.85)	0.36	4.24 (0.64)	4.47 (0.61)	0.01
Contraceptive methods	3.68 (0.59)	3.77 (0.59)	0.44	4.14 (0.55)	4.44 (0.63)	0.000	4.56 (0.56)	4.71 (0.49)	0.16	4.2 (0.54)	4.6 (0.48)	0.000
Sexual and reproductive rights	3.41 (0.59)	3.55 (0.75)	0.31	3.94 (0.63)	4.13 (0.54)	0.15	3.42 (0.49)	3.79 (0.68)	0.04	4.07 (0.58)	4.55 (0.55)	0.000
Rights violations	3.17 (0.77)	3.21 (0.78)	0.61	3.07 (0.92)	3.18 (0.82)	0.41	3.1 (0.73)	3.17 (0.82)	0.98	4.08 (0.78)	4.35 (0.68)	0.01
Gender-based violence	3.3 (0.85)	3.27 (0.93)	0.99	3.23 (1)	3.5 (1.11)	0.1	3.64 (1.08)	3.91 (1.02)	0.2	4.02 (0.93)	4.34 (0.72)	0.04
Overall score	23.84 (2.77)	24.41 (3.11)	0.1	24.9 (3.04)	26.6 (3.39)	0.000	25.27 (3.11)	26.73 (3.8)	0.07	28.26 (3.5)	31.14 (3.58)	0.000

SD, standard deviation; *p*, p-value (Wilcoxon signed-rank test).

* Wilcoxon test; *p* = corresponding p-value. *p* values < 0.05 were considered statistically significant.

### 3.3 Satisfaction levels

**Figure 1:**
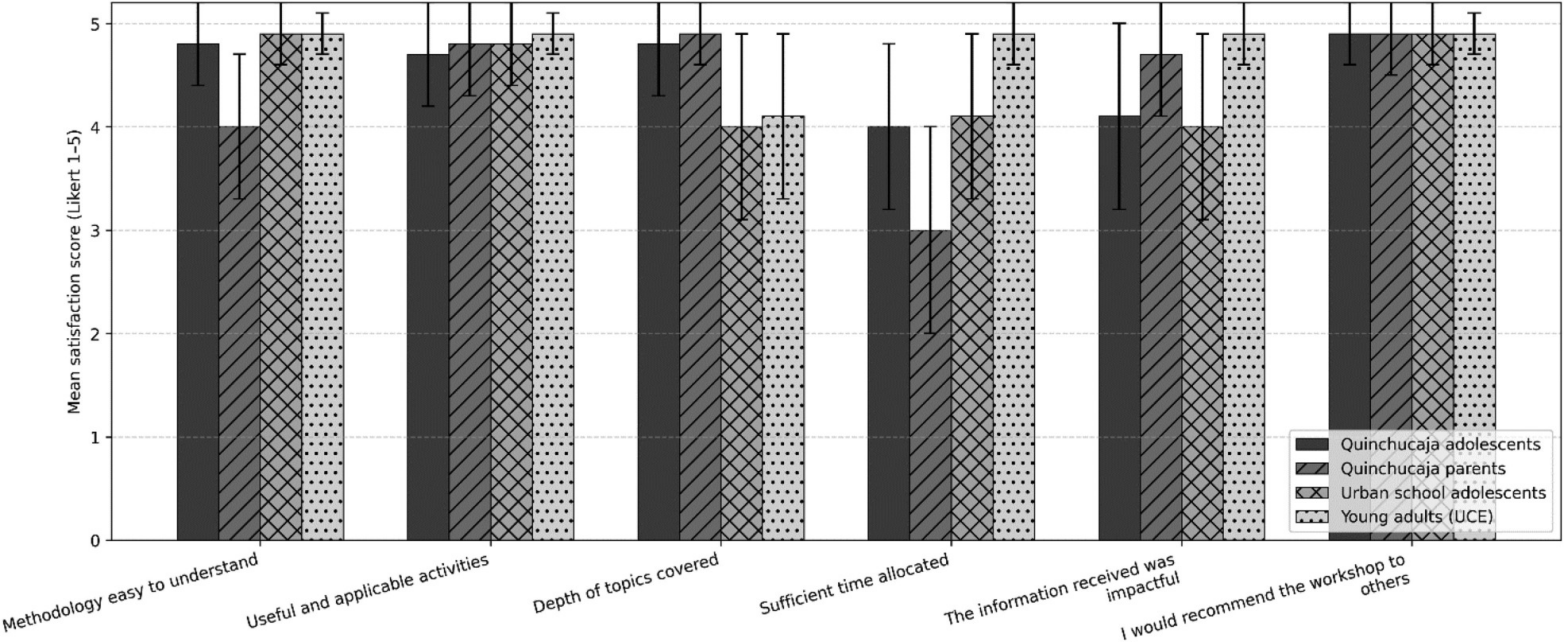
Satisfaction survey across the four intervention groups. Bars represent mean satisfaction scores with standard deviation, measured using a 5-point Likert scale (1 = very low, 5 = very high), across six evaluation domains: clarity of the methodology, usefulness of activities, depth of topics covered, adequacy of time allocation, perceived impact of the information received, and willingness to recommend the workshop. Results are presented separately for Quinchucaja adolescents, Quinchucaja parents, urban school adolescents, and young adults enrolled at the Central University of Ecuador (UCE).

Participant satisfaction was assessed using a 5-point Likert-scale questionnaire across six evaluation domains. Mean satisfaction scores were generally high among adolescents and young adults, while lower scores were observed among parents, particularly regarding time allocation (Figure 1).

### 3.4 Training impact on facilitators

**Figure 2:**
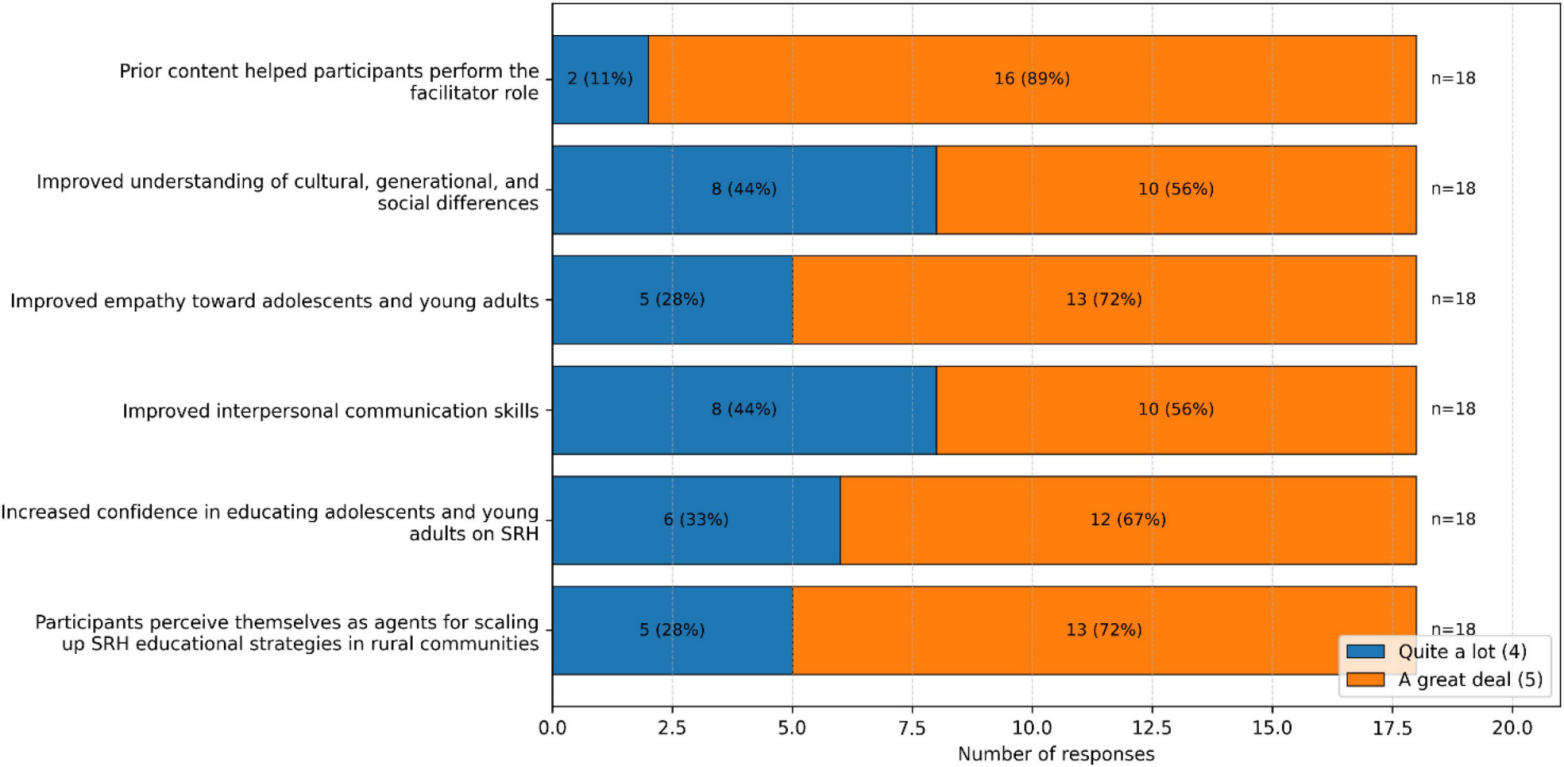
Perceived improvement in facilitators’ skills. Bars show the distribution of high agreement responses (*Quite a lot* (4) and *A great deal* (5) on a 5-point Likert scale, expressed as frequencies and percentages (*n* = 18). Items reflect perceived preparedness, empathy, communication skills, confidence in delivering SRH education, and perceived capacity to support scale-up of educational strategies.

Eighteen Obstetrics undergraduate students participated as peer facilitators and completed post-intervention evaluations. High levels of agreement (“Quite a lot” and “A great deal”) were reported across all assessed domains, including preparedness for the facilitator role, empathy toward adolescents, interpersonal communication skills, and confidence in delivering sexual and reproductive health education (Figure 2).

Student narratives, summarized in Table 4, highlight a positive appraisal of the facilitator role, underscoring its contribution to vocational reaffirmation, the broadening of professional horizons, and comprehensive training. Within the subcategory Vocational reaffirmation, participants reported that the experience strengthened their commitment to pursuing an empathetic and engaged professional practice, recognizing that “being able to educate others requires a great deal of vocation and confidence,” or that “it helped me envision the kind of professional I want to be: approachable, empathetic, and capable of adapting to people's diverse realities.”

**Table 4:** Experiences and perceptions of facilitators—university students.

Categories and subcategories	*Verbatim Quotations*
1. Role as facilitators	
- Assumed an active role as SRH educators	*“We became reference figures, and that changed the dynamic with the adolescents—they listened to us.”*
- Adaptation of the methodology to the context	*“We had to explain using our own examples and our own words.”*
- Management of communicative and pedagogical challenges	*“It was difficult at first, but then we learned how to handle uncomfortable questions.”*
2. Professional impact	
- Consolidation of technical competencies	*“It helped me speak with confidence, even when addressing adults.”*
- Development of interpersonal and leadership skills	*“It improved my ability to listen, understand, and refrain from judging.”*
- Motivation for future community health interventions	*“Now I want to work in rural areas, because I saw that real change is possible.”*
3. Personal impact	
- Revaluation of their role as advanced students	*“I realized that everything we learned truly makes sense when you put it into practice.”*
- Strengthening of self-confidence and self-esteem	*“Seeing how people responded made me feel that I was genuinely doing something useful.”*
- Transformation of attitudes toward sociocultural diversity	*“I learned to respect other ways of thinking, even if I do not share them.”*

SRH, sexual and reproductive health.

The subcategory Expansion of the professional field captures perceptions reflecting openness to new avenues of professional practice beyond traditional clinical settings, such as “it made me realize that my career has many paths” and “I became aware that there is a vast field of work.” Finally, the subcategory Contribution to comprehensive training indicates that the intervention fostered key practical skills, including effective communication, adaptability, and engagement with diverse populations: “you learn how to manage yourself and respond by adapting to the environment and the people you are working with.”

## 4 Discussion

Interviews with key informants indicated that, although the original strategy was positively valued, it required both structural and contextual adjustments. These included clearer definition of learning objectives for each station, revision of content addressing complex topics (e.g., HIV, abortion, and sexual violence), adaptation of graphic materials, and the explicit integration of referral pathways and institutional support networks for situations involving abuse. On this basis, an adapted version was consolidated, featuring fewer stations, more realistic time allocations, clearly defined opening and closing key messages, the incorporation of audiovisual and digital tools, and updated instructional guidelines to support differentiated participatory dynamics according to the target group. From a rights-based and intercultural education perspective, these adaptations respond to the need to ensure that SRH educational interventions are accessible, culturally appropriate, and aligned with adolescents’ lived realities, particularly in contexts marked by structural inequities (12, 13). The emphasis on selecting young facilitators with prior training in SRH and reduced generational gaps relative to participants is consistent with evidence highlighting the value of co-design processes and participatory approaches to enhance cultural relevance and acceptability of adolescent SRH interventions (21–26). This finding is also coherent with principles of critical pedagogy, which emphasize horizontal dialogue and the co-construction of knowledge as mechanisms for meaningful learning (16). It also aligns with studies documenting the potential of peer education to strengthen knowledge, attitudes, and self-efficacy in sexual and reproductive health when supported by adequate technical preparation and a clear methodological structure (27).

In Latin America, programs frequently face gaps between knowledge and action, driven by factors such as limited administrative support, high workloads, and cultural resistance (28–31). Accordingly, our findings underscore the importance of articulating local actors and integrating institutional support networks as a prerequisite for workshop implementation, thereby reinforcing response capacity and fostering environments that legitimize the exercise of sexual and reproductive rights. From a rights-based framework, the explicit incorporation of referral pathways and institutional coordination can be interpreted as a key mechanism to operationalize sexual and reproductive rights beyond information provision, enabling concrete responses to situations of violence or rights violations. Several studies indicate that successful interventions incorporate coordination with local stakeholders (31–33), which strengthens social ownership of strategies, particularly when combined with social incentives and institutional recognition (28, 34). This is further reinforced by the interdisciplinary approach of Rurankapak, which aligns with comprehensive education models integrating health sciences, pedagogy, and human rights, as described in experiences from Mexico, Peru, and Chile (35–37). The integration of biological, psychological, and social perspectives promotes a holistic understanding of sexuality and contrasts with reductionist approaches centered solely on reproductive biology (38, 39), advancing a more equitable, participatory, and empowerment-oriented model of sexual education consistent with principles of Latin American emancipatory education.

The incorporation of digital platforms and audiovisual resources is also recommended, as such tools have demonstrated potential to expand the reach of sexual education (27). Effective programs such as COMPAS in Colombia, as well as technology-based adaptations implemented in Ecuador and Nicaragua, show that culturally sensitive and participatory approaches supported by accessible technologies and local narratives enhance adolescent identification and engagement (40–42). However, as highlighted by experiential learning theory, technology alone does not generate meaningful learning without opportunities for active participation and reflection (17). At the same time, as technology does not replace face-to-face accompaniment or the group dynamics required to promote critical reflection (34, 43–46), methodological hybridization preserves an essential human component that ensures value transmission and contextual understanding of sexuality.

Several Rurankapak stations aim to promote attitudinal change, curiosity, and skills for seeking reliable information. Although pre- and posttest results showed statistically significant improvements—particularly among young adults and rural/Indigenous secondary school students in topics related to sexuality and contraception—the assessment instrument focused primarily on declarative knowledge. The greater improvements observed among young adults may be partially explained by higher baseline educational exposure and familiarity with participatory learning environments, while lower gains among parents likely reflect cultural resistance and limited familiarity with dialogical methodologies. The brevity of each station, the limited overall workshop duration, and the breadth of topics constrain the detection of cognitive variation through structured questionnaires. Consequently, results should be interpreted as a partial approximation of intervention impact and support the need to refine station-specific learning objectives and incorporate evaluation tools aligned with the formative logic of the methodology. Evidence indicates that participatory and culturally adapted sexual education programs improve knowledge and competencies (32, 33, 43, 47, 48), while evaluations based solely on knowledge tend to overestimate cognitive gains and fail to capture decision-making in real-life contexts (49). Studies using case-based and scenario-based assessments show that meaningful learning is better reflected in problem-solving and critical reflection than in closed-ended responses (38, 39, 50). This reinforces the need for future evaluations of Rurankapak to incorporate indicators of attitudes, self-efficacy, and practical application of knowledge, in addition to information retention.

Beyond adaptability to diverse sociocultural contexts, the methodology also strengthened facilitator competencies, mirroring other Latin American experiences in which comprehensive educator training—such as that observed in the CERCA project in Bolivia, Ecuador, and Nicaragua (47, 51)—improved facilitator confidence and clarity, positively influencing workshop effectiveness. Consistent with peer education theory and critical pedagogy, the use of facilitators with reduced generational distance may have contributed to the creation of more horizontal learning spaces, fostering empathy, communication skills, and confidence in addressing sensitive SRH topics (16, 18). Recent literature emphasizes that educator training is among the most critical determinants of intervention sustainability (34–37, 48), and that success depends more on continuous accompaniment processes than on isolated training events (28, 33). However, this relationship is not linear: in several Latin American contexts, teachers and promoters recognize program value but fail to apply content in everyday practice (32, 39), demonstrating that training without institutional backing or structural incentives tends to generate only partial impacts.

In the Latin American context, SRH literacy has been associated with reductions in adolescent pregnancy, evidencing a dose–response relationship between knowledge and prevention of both first and repeat pregnancies (52). Nevertheless, multiple reviews (32, 34, 35, 53, 54) caution that such results are concentrated mainly in urban settings, with persistent gaps in rural and Indigenous areas. Structural barriers—including poverty, distance to services, stigma, and educational inequality—continue to limit effective access to SRH education and care (33, 36, 37). This aligns with our findings, where knowledge improvements did not translate into homogeneous changes across groups, and where parents and rural students reported greater difficulties in comprehension and internalization of content. These disparities further underscore the relevance of culturally responsive and rights-based educational strategies to address persistent inequities in SRH outcomes. Additionally, Ecuadorian studies have shown that setbacks in SRH policies—such as reduced funding or institutional fragmentation—correlate with increases in adolescent birth rates in Indigenous communities (55), confirming the fragility of gains in the absence of sustained political continuity (29, 35).

While Rurankapak successfully adapted to heterogeneous contexts while preserving its pedagogical core, the parental component proved more challenging. Latin American studies document that even parents with adequate educational backgrounds often avoid discussing sexuality due to fear of judgment, discomfort, or beliefs that such topics are “inappropriate” for certain ages, perpetuating intergenerational silence. From a critical pedagogy perspective, these findings suggest that parental engagement requires not only information delivery but also dialogical spaces that challenge entrenched norms and facilitate collective reflection (16). This cultural tension helps explain why knowledge gains do not always translate into sustained communicational change. Our findings therefore reinforce the need to design materials and activities that not only transmit information but also challenge traditional imaginaries and legitimize open dialogue between parents and children. Evidence suggests that visual formats, testimonial narratives from community members, and exercises grounded in everyday experiences foster adherence and empathy, facilitating the overcoming of cultural and emotional resistance (39, 56–58). Parental involvement remains a key determinant for sustaining learning and consolidating favorable communicational environments around sexuality and affectivity (43, 56). Nonetheless, beyond literacy levels, the main barriers are not solely related to content comprehension but to the weight of taboo, stigma, and restrictive cultural norms that hinder addressing these topics within the family sphere.

### 4.1 Strengths

The Rurankapak strategy demonstrated a strong capacity to adapt to diverse educational and population settings, standing out for its flexibility in responding to contextual differences. The original guide was explicitly designed to be modified according to each community's cultural and social characteristics while preserving its educational essence. This flexibility allowed adjustment of language and examples according to educational level, adaptation of participatory dynamics to urban and rural contexts, and maintenance of the seven thematic stations while optimizing participant flow.

The project successfully articulated a multidisciplinary approach integrating health, education, and rights, combining perspectives from Health Sciences (scientific approaches to contraception, menstrual health, and prevention), Social Sciences (social and cultural determinants of sexuality), Human Rights (a sexual and reproductive rights–based approach), Pedagogy (participatory methodology adapted to different educational levels), and Psychology (emotional and relational dimensions of sexuality). This integration enabled a comprehensive approach that goes beyond traditional biologically centered sexual education.

Data analysis demonstrated a high level of methodological rigor, including systematic processing with digitization, tabulation, and cleaning of 8,050 data points; robust statistical methods using non-parametric tests (Wilcoxon) appropriate for Likert-type scales; transparent handling of missing data with consistent imputation criteria; multidimensional analysis by thematic stations and global score; and methodological triangulation combining quantitative results with satisfaction surveys.

To situate our contribution within the existing evidence base, we reviewed peer-reviewed studies on SRH education and adolescent-focused interventions in Ecuador and Latin America. In Ecuador, prior research has examined mechanisms and barriers for adolescent-friendly SRH services, as well as acceptability of technology-mediated SRH risk-reduction approaches among adolescents (34). Related regional evidence has also documented peer-oriented delivery models and systematic cultural adaptation of school-based sexual health promotion programs in Latin America (31). However, to our knowledge, no peer-reviewed study in Ecuador has evaluated a participatory, station-based “circuit” methodology delivered by peer facilitators and implemented across four distinct sociocultural contexts, as in the present study. This gap underscores the value of documenting not only preliminary outcomes, but also the practical conditions of applicability and acceptability when implementing culturally responsive, peer-facilitated SRH education strategies in heterogeneous settings.

### 4.2 Limitations

The quasi-experimental design without a concurrent control group limits the ability to attribute observed changes exclusively to the adapted Rurankapak intervention. Although the pre–post approach enabled the assessment of preliminary changes in knowledge and acceptability across groups, causal inferences cannot be established. While implementing the intervention across four distinct sociocultural contexts allowed for comparative benchmarking of applicability and consistency of patterns, this design does not substitute for a true control arm. Future studies should therefore employ more rigorous designs, such as cluster-randomized or stepped-wedge trials with explicit comparison conditions, to strengthen causal inference and estimate intervention effects more precisely.

The study was exploratory and feasibility-oriented; consequently, no *a priori* sample size calculation was performed. Sample sizes were determined by institutional feasibility and participant availability, which may have limited statistical power to detect changes across all groups, particularly among parents and rural participants. As a result, some non-significant findings should be interpreted with caution. Future research should incorporate formal sample size estimation and larger, balanced samples to improve power and enable subgroup analyses.

Implementation was conducted in specific sociocultural contexts, including rural, Indigenous, and urban-marginal settings in Ecuador. Although Rurankapak is intentionally designed to be adaptable, the context-specific nature of the intervention may limit generalizability to other regions or populations with different cultural, educational, or institutional characteristics. Multi-site studies across diverse geographic and policy environments are warranted to assess transferability and scalability.

The quantitative evaluation focused primarily on knowledge acquisition and participant satisfaction using Likert-type self-reported measures, which are susceptible to social desirability bias and may not fully capture deeper changes in attitudes, behaviors, or real-life decision-making related to sexual and reproductive health. Moreover, the short duration of the intervention relative to the breadth of topics covered may have constrained the magnitude of measurable cognitive change. Future studies should incorporate longitudinal follow-up and complementary outcome measures—such as scenario-based assessments, behavioral indicators, and self-efficacy scales—to better reflect meaningful learning and sustained impact.

Finally, facilitator-related factors, including prior training, motivation, and interpersonal skills, may have influenced participant engagement and learning outcomes. Although standardized training procedures and methodological guides were used to promote fidelity of implementation, variability in facilitation dynamics cannot be entirely ruled out. Future research should examine facilitator effects more systematically and explore the added value of extended training, supervision, and fidelity monitoring mechanisms.

### 4.3 Areas for improvement

To address identified limitations, the development of more didactic materials for parents is recommended, including visually oriented guides with reduced text and increased iconography, testimonial videos featuring parents from the same community, exercises based on real family situations, glossaries using everyday language, and preliminary familiarization sessions with the methodology.

In parallel, incorporating a greater number of local examples could strengthen identification with the content. This includes community-based narratives and cases, local geographic and cultural references, recognizable characters and situations, adaptation of terminology to local dialectal variants, and explicit linkage of content to sexual and reproductive health (SRH) issues identified within each community.

At the methodological level, future implementations and evaluations would benefit from more explicit reporting of qualitative procedures, including sampling strategies, analytic steps, and criteria used to determine data saturation. Clear documentation of these processes would enhance transparency, credibility, and the reproducibility of qualitative findings across settings.

Looking ahead, subsequent studies should move toward longitudinal designs with follow-up assessments to evaluate sustained knowledge retention and behavioral outcomes. The inclusion of explicit comparison groups would strengthen causal inference, while the use of complementary evaluation tools—such as measures of attitudes, self-efficacy, and real-life decision-making—would provide a more comprehensive assessment of intervention impact. Research conducted in additional geographic and cultural contexts would further inform the scalability and broader applicability of the adapted Rurankapak strategy.

## 5 Conclusions

This study demonstrates that interinstitutional collaboration between academia and international organizations specialized in sexual and reproductive health (SRH) constitutes a strategic pillar for the design, adaptation, and evaluation of educational methodologies such as “Rurankapak.” This joint effort enabled the integration of technical expertise, field experience, and critical interpretation of sociocultural contexts, resulting in a methodological version that is clearer, more contextually relevant, and operationally feasible. The experience suggests that sexual education programs in Latin America should combine theoretical assessment with practical case analysis, integrate qualitative and quantitative methodologies to better understand target populations, and adapt content to sociocultural and age-specific contexts, ensuring that interventions respond to real needs and to the particularities of rural, urban, and historically marginalized communities.

Complementarily, the findings indicate that university students enrolled in health-related programs—particularly Obstetrics—can serve as highly effective peer facilitators by reducing generational gaps and fostering environments of trust for addressing sensitive SRH topics. This role simultaneously strengthens their technical competencies and soft skills—such as communication, empathy, and an intercultural perspective—while promoting more comprehensive, rights-based, and participatory interventions among adolescents and young people. The integration of digital tools and active learning methodologies in the adapted version of “Rurankapak” emerges as a promising pathway to enhance comprehension, retention, and application of knowledge, especially among young populations and in rural or highly socially vulnerable contexts.

## Data Availability

The raw data supporting the conclusions of this article will be made available by the authors, without undue reservation.
