# Supplementary material for: Evaluation of the contextualized sexual and reproductive health educational strategy “Rurankapak”: a mixed-methods quasi-experimental study among adolescents and young people in Ecuador
Source: Front Reprod Health. 2026 Feb 19;8:1783094. doi: 10.3389/frph.2026.1783094 (PMC12960122; doi:10.3389/frph.2026.1783094)
Supplement: Supplementary file 1 [file Table1.docx]

**CODIGO: ….…………..……**

**TALLER-Proyecto:** **Mejorando la Salud Sexual y Reproductiva: adaptación de una Metodología Educativa para Adolescentes y Padres en un Colegio Rural Ecuatoriano**

Lee cada afirmación y marca la opción que mejor refleje tu opinión o conocimiento. No te preocupes que no hay respuestas correctas o incorrectas.

**GENERO:** Femenino ( ) Masculino ( ) Otro ( ) **Edad…………**

**ETNIA: te consideras,** Mestizo ( ) Indígena ( ) Blanco ( ) Montubio ( ) Otro ( )

**ESTACIÓN 1:**

**E1.1. Algunas personas creen que una mujer no puede quedar embarazada si tiene relaciones sexuales durante su periodo menstrual. ¿Qué tan de acuerdo estás con esta afirmación?**

a) Totalmente de acuerdo

b) Bastante de acuerdo

c) Algo de acuerdo

d) Poco de acuerdo

e) Nada de acuerdo

**E1.2. Cuando tienes dudas sobre cómo prevenir un embarazo no deseado, ¿qué tan probable es que consultes más información en fuentes tales como profesionales de salud, profesores que conozcan el tema o enlaces de internet de fuentes confiables?**

1. Muy probable, seguramente buscaré más información
2. Bastante probable
3. Algo probable
4. Poco probable
5. Nada probable, no consultaré información adicional

**E1.3.** **Algunas personas creen que una mujer no puede quedar embarazada si orina después de tener relaciones sexuales. ¿Qué tan de acuerdo estás con esta afirmación?**

a) Totalmente de acuerdo

b) Bastante de acuerdo

c) Algo de acuerdo

d) Poco de acuerdo

e) Nada de acuerdo

**E1.4. Si un amigo te habla de una afirmación sobre cómo evitar el embarazo no deseado y que no conocías, ¿qué tan probable es que verifiques la información antes de creerla?**

a) Muy probable

b) Bastante probable

c) Algo probable

d) Poco probable

e) Nada probable

**ESTACIÓN 2**

**E2.1. ¿Consideras que la sexualidad abarca más que solo el sexo o el aparato sexual?**

a) Totalmente de acuerdo

b) Bastante de acuerdo

c) Algo de acuerdo

d) Poco de acuerdo

e) Nada de acuerdo

**E2.2. ¿consideras que dar un abrazo o regalar una flor a tu pareja, podría considerarse como un componente de la sexualidad?**

a) Muy probable

b) Bastante probable

c) Algo probable

d) Poco probable

e) Nada probable

**E2.3. consideras que la orientación sexual de una persona no debería estar determinada por la forma en que la iglesia o la comunidad la juzguen.** a) Totalmente de acuerdo
 b) Bastante de acuerdo
 c) Algo de acuerdo
 d) Poco de acuerdo
 e) Nada de acuerdo

**ESTACIÓN 3**

**E3.1. consideras que el conocimiento del ciclo menstrual es útil para prevenir un embarazo.**a) Totalmente de acuerdo
b) Bastante de acuerdo
c) Algo de acuerdo
d) Poco de acuerdo
e) Nada de acuerdo

**E3.2. Jessica está menstruando y su madre le dice que “está enferma” y no puede realizar ciertas actividades como bañarse. ¿Qué tan de acuerdo estás con esta creencia?**a) Totalmente de acuerdo
b) Bastante de acuerdo
c) Algo de acuerdo
d) Poco de acuerdo
e) Nada de acuerdo

**E3.3. ¿Qué tan probable es que hables abiertamente sobre la menstruación con otras personas para promover información correcta?**

a) Muy probable

b) Bastante probable

c) Algo probable

d) Poco probable

e) Nada probable

**ESTACIÓN 4**

**E4.1. El novio de Andrea afirma que los métodos anticonceptivos son solo para ser usados por mujeres. ¿Qué tan de acuerdo estás con esta afirmación?**a) Totalmente de acuerdo
b) Bastante de acuerdo
c) Algo de acuerdo
d) Poco de acuerdo
e) Nada de acuerdo

**E4.2. ¿Qué tan de acuerdo estás con la afirmación: "Los métodos anticonceptivos de duración larga, ¿tales como inyecciones, DIU o implantes tienen ventajas adicionales como ser más discretos y una buena opción para adolescentes o jóvenes?**

a) Totalmente de acuerdo
b) Bastante de acuerdo
c) Algo de acuerdo
d) Poco de acuerdo
e) Nada de acuerdo

**E4.3. ¿Qué tan de acuerdo estás con esta afirmación? Los métodos anticonceptivos de barrera, como el preservativo masculino y el preservativo femenino, son la mejor opción para prevenir infecciones de transmisión sexual y prevenir embarazos no deseados.**

a) Totalmente de acuerdo
b) Bastante de acuerdo
c) Algo de acuerdo
d) Poco de acuerdo
e) Nada de acuerdo

**E.4.4. ¿Qué tan probable es que recomiendes a un amigo o amiga que se informe sobre anticonceptivos con un profesional de salud?**

a) Muy probable

b) Bastante probable

c) Algo probable

d) Poco probable

e) Nada probable

**ESTACIÓN 5**

**E5.1. Jessica, una adolescente, intenta comprar anticonceptivos en una farmacia, pero se los niegan porque es menor de edad. ¿Consideras que esta situación es una vulneración de sus derechos sexuales y reproductivos?**

a) Totalmente de acuerdo
b) Bastante de acuerdo
c) Algo de acuerdo
d) Poco de acuerdo
e) Nada de acuerdo

**E5.2. A una estudiante embarazada le impiden continuar con sus estudios. ¿Qué tan de acuerdo estás con que esta situación?**

a) Totalmente de acuerdo
b) Bastante de acuerdo
c) Algo de acuerdo
d) Poco de acuerdo
e) Nada de acuerdo

**E5.3. Para que un adolescente pueda acceder a un método anticonceptivo en un centro de salud, debe acudir obligatoriamente acompañado por sus padres. ¿Qué tan de acuerdo estás con esta afirmación?**a) Totalmente de acuerdo
b) Bastante de acuerdo
c) Algo de acuerdo
d) Poco de acuerdo
e) Nada de acuerdo

**E5.4. Si tú o alguien cercano enfrentara una situación de vulneración de derechos educativos o sexuales, ¿qué tan probable es que busques apoyo en una red de bienestar estudiantil o con un docente de confianza?**

a) Muy probable

b) Bastante probable

c) Algo probable

d) Poco probable

e) Nada probable

**ESTACIÓN 6**

**E6.1. Cumandá, una adolescente de 15 años, queda embarazada y quiere seguir estudiando, pero no se le permite. ¿Qué tan de acuerdo estás en que debe recibir apoyo para continuar con su educación?**

a) Totalmente de acuerdo

b) Bastante de acuerdo

c) Algo de acuerdo

d) Poco de acuerdo

e) Nada de acuerdo

**E6.2. ¿Consideras que las dificultades económicas, sociales y familiares influyen para que una adolescente quede embarazada?**

a) Totalmente de acuerdo

b) Bastante de acuerdo

c) Algo de acuerdo

d) Poco de acuerdo

e) Nada de acuerdo

**E6.3. ¿Qué tan de acuerdo estás con la afirmación?: La violencia hacia las mujeres en las relaciones de pareja puede afectar su salud sexual y reproductiva con la aparición de embarazos no deseados o la transmisión de infecciones.**a) Totalmente de acuerdo
b) Bastante de acuerdo
c) Algo de acuerdo
d) Poco de acuerdo
e) Nada de acuerdo

**E6.4. En qué medida consideras que las adolescentes embarazadas no requieren mayor apoyo pues ella se lo buscó, y como ya sabía a lo que se metía ahora debe asumir las consecuencias**

a) Totalmente de acuerdo

b) Bastante de acuerdo

c) Algo de acuerdo

d) Poco de acuerdo

e) Nada de acuerdo

**ESTACIÓN 7**

**E7.1. Una persona es fotografiada sin su consentimiento y sus fotos íntimas son difundidas. ¿Qué tan de acuerdo estás en que esta situación representa un tipo de violencia?**

a) Totalmente de acuerdo

b) Bastante de acuerdo

c) Algo de acuerdo

d) Poco de acuerdo

e) Nada de acuerdo

**E7.2. Si tú o alguien cercano enfrentara una situación de violencia, ¿qué tan probable es que busques apoyo en una red de bienestar estudiantil o con un docente de confianza?**

a) Muy probable

b) Bastante probable

c) Algo probable

d) Poco probable

e) Nada probable

**E7.3. Sofía, una joven de 18 años, mantiene una relación con un hombre de 24 que financia sus estudios. Aunque desea terminarla, él y su familia la presiona para continuar debido al apoyo económico que él hombre da a ella y su familia. ¿Qué tan de acuerdo estás en que esta situación representa una forma de violencia?**

a) Totalmente de acuerdo

b) Bastante de acuerdo

c) Algo de acuerdo

d) Poco de acuerdo

e) Nada de acuerdo
